# Scoping review of indications for robotic ventral mesh rectopexy: addressing variability in patient selection

**DOI:** 10.1007/s00384-025-04893-y

**Published:** 2025-04-25

**Authors:** Ugo Grossi, Simona Ascanelli, Nicola de’Angelis, Eugenia Sinatti, Angelo D’Ovidio, Carlo Alberto Schena, Gaetano Gallo, Eleonora Mollica, Antonino Lauria, Alvise Frasson, Fabrizio Vittadello, Gabriele Naldini

**Affiliations:** 1 Department of Surgery, Oncology and Gastroenterology – DiSCOG, University of Padova, Padua, Italy; 2 Surgery Unit 2, Regional Hospital Treviso ‘Cittadella Della Salute’, Treviso, Italy; 3 Unit of Robotic and Minimally Invasive Digestive Surgery, Department of Surgery, Ferrara University Hospital Arcispedale Sant’Anna, Ferrara, Italy; 4 Department of Translational Medicine and LTTA Centre, University of Ferrara, Ferrara, Italy; 5 General Surgery Unit, Azienda ULSS, Serenissima, Mestre, Italy; 6 Department of Surgery, Sapienza University of Rome, Rome, Italy; 7 Department of Surgery, Hospital Sant’ Antonio, University of Padova, Padua, Italy; 8 Proctology and Pelvic Floor Clinical Center, Cisanello University Hospital, Pisa, Italy

**Keywords:** Ventral mesh rectopexy, Robotic, Indications, Surgery, Scoping review

## Abstract

**Introduction:**

Ventral mesh rectopexy (VMR) has gained popularity as a surgical solution for rectal prolapse. However, significant variability exists in patient selection criteria, preoperative evaluation, and reporting standards. This scoping review analyzes indications for robotic VMR (RVMR) and highlights areas requiring further standardization.

**Methods:**

The review was conducted according to PRISMA-ScR guidelines. Comprehensive searches of PubMed, Scopus, and Web of Science were completed through December 4, 2024. Studies reporting on RVMR were screened in a three-step process, with disagreements resolved by consensus. Key data extracted included patient demographics, indications, preoperative workup, and surgical details. Superseded studies, reviews, and non-relevant articles were excluded.

**Results:**

Of 783 articles identified, 24 studies comprising 930 patients met inclusion criteria. External rectal prolapse was the most common indication (47%), followed by intussusception (38%), rectocele (9%), combined abnormalities (5%), and enterocele (1%). Preoperative imaging was inconsistently reported, with only 67% of studies describing imaging protocols. Symptom-based indications using standardized scoring systems were rare (17%). Synthetic mesh was used in 87% of cases. RVMR showed favorable functional outcomes, with low recurrence and complication rates.

**Conclusions:**

Significant heterogeneity exists in indications and preoperative evaluation for RVMR, limiting comparability across studies. While evidence supports its safety and efficacy, future research should focus on standardizing selection criteria and evaluating long-term outcomes.

**Supplementary Information:**

The online version contains supplementary material available at 10.1007/s00384-025-04893-y.

## Introduction

Structural abnormalities of the anorectum are diverse, encompassing a range of conditions that may significantly impair bowel function and quality of life [1]. The evaluation of these abnormalities often relies on dynamic imaging modalities, such as defecography, which provide critical insights into functional and anatomical disturbances in patients with pelvic floor disorders [2].

Since the introduction of laparoscopic ventral mesh rectopexy (LVMR) by D’Hoore in 2004 [3], the robotic approach has emerged as a promising alternative, offering potential advantages in precision and ergonomics [4]. Indeed, robotic-assisted surgery has been shown to enhance precision, dexterity, and overall surgical accuracy, particularly in complex steps like mesh fixation and rectovaginal dissection, offering superior ergonomics compared to conventional laparoscopy [5]. Despite these potential advantages, robotic VMR (RVMR) is associated with certain disadvantages, including longer operative times and higher costs compared to conventional laparoscopy.

However, despite its growing popularity, the indications for VMR appear to be heterogeneous across studies, with variations in patient selection criteria and clinical scenarios. Our previous systematic review on hitching procedures for obstructed defecation syndrome revealed significant heterogeneity in patient selection criteria and terminology [6]. Similarly, the diagnostic pathways and inclusion criteria for surgical intervention were highly variable, with some studies focusing on complex multicompartment pelvic floor disorders while others targeted isolated anatomical abnormalities. These inconsistencies in patient selection not only complicate the synthesis of evidence but also raise concerns about the generalizability of findings. However, at that time, data on RVMR were still scarce and in their infancy, thus precluding any detailed analysis of indications or practices specifically related to RVMR. This scoping review was therefore undertaken to address this gap by focusing exclusively on RVMR. By synthesizing the current evidence, we sought to provide a comprehensive overview of the rationale for RVMR and highlight areas requiring further clarification or standardization.

## Methods

### Study design

This review was conducted in accordance with the guidelines for the Preferred Reporting Items for Systematic reviews and Meta-Analyses extension for Scoping Reviews (PRISMA-ScR) [7].

### Search strategy and screening

We conducted a comprehensive search of the literature using PubMed, Scopus, and Web of Science through December 4 th 2024. The search strategy was carefully designed to capture all relevant studies that reported on patients undergoing RVMR (Appendix 1).

Once the search was completed, all retrieved records were subjected to a three-step screening process. Two reviewers (UG and ES) independently screened all titles, abstracts, and full-texts. Initially, titles were reviewed to exclude clearly irrelevant studies. Subsequently, abstracts of the remaining records were assessed to determine their relevance. Finally, full-text articles of potentially eligible studies were thoroughly reviewed to confirm their inclusion.

Throughout this process, any disagreements or discrepancies regarding study inclusion were resolved through discussion and consensus.

### Eligibility criteria

To capture the full scope of the literature, no restrictions were applied to the study design. This inclusive approach allowed to consider a variety of sources, including case reports, case series, cohort studies, and randomized controlled trials, to ensure a comprehensive understanding of the indications for RVMR across diverse clinical contexts.

Studies were excluded if they represented superseded series, i.e., where the same patient cohort was included in a later publication from the same institution with a larger sample size. Editorials, commentaries, and opinion pieces were also excluded. Similarly, studies that focused on surgical procedures associated with RVMR (e.g., combined sacrocolpopexy) were not considered for inclusion. Additionally, we excluded studies that mentioned RVMR but did not provide specific data about this procedure. Finally, reviews and meta-analyses were also excluded, but only after ensuring that they did not reference studies or data that needed to be added to our search.

### Data extraction

From each included study, we systematically extracted key information. These included whether the study was single-center or multicenter, the total number of patients included, and demographic data such as patient age and proportions of female/male patients, the study design, and duration (months). Detailed clinical and procedural data were also collected, including the prevalence of specific anorectal abnormalities such as external (ERP) or internal rectal prolapse (IRP), rectocele, enterocele, and/or combined abnormalities. Additionally, we extracted information on other indications for RVMR, and data on imaging modalities (e.g., X-ray or magnetic resonance imaging [MRI] defecography) and other preoperative tests used to evaluate patients. We also noted the use of preoperative scoring systems for symptom severity or functional assessment.

Details about the surgical procedure were extracted, including the type of robotic system employed and the type of mesh used (synthetic or biologic).

## Results

### Study selection

**Fig. 1:**
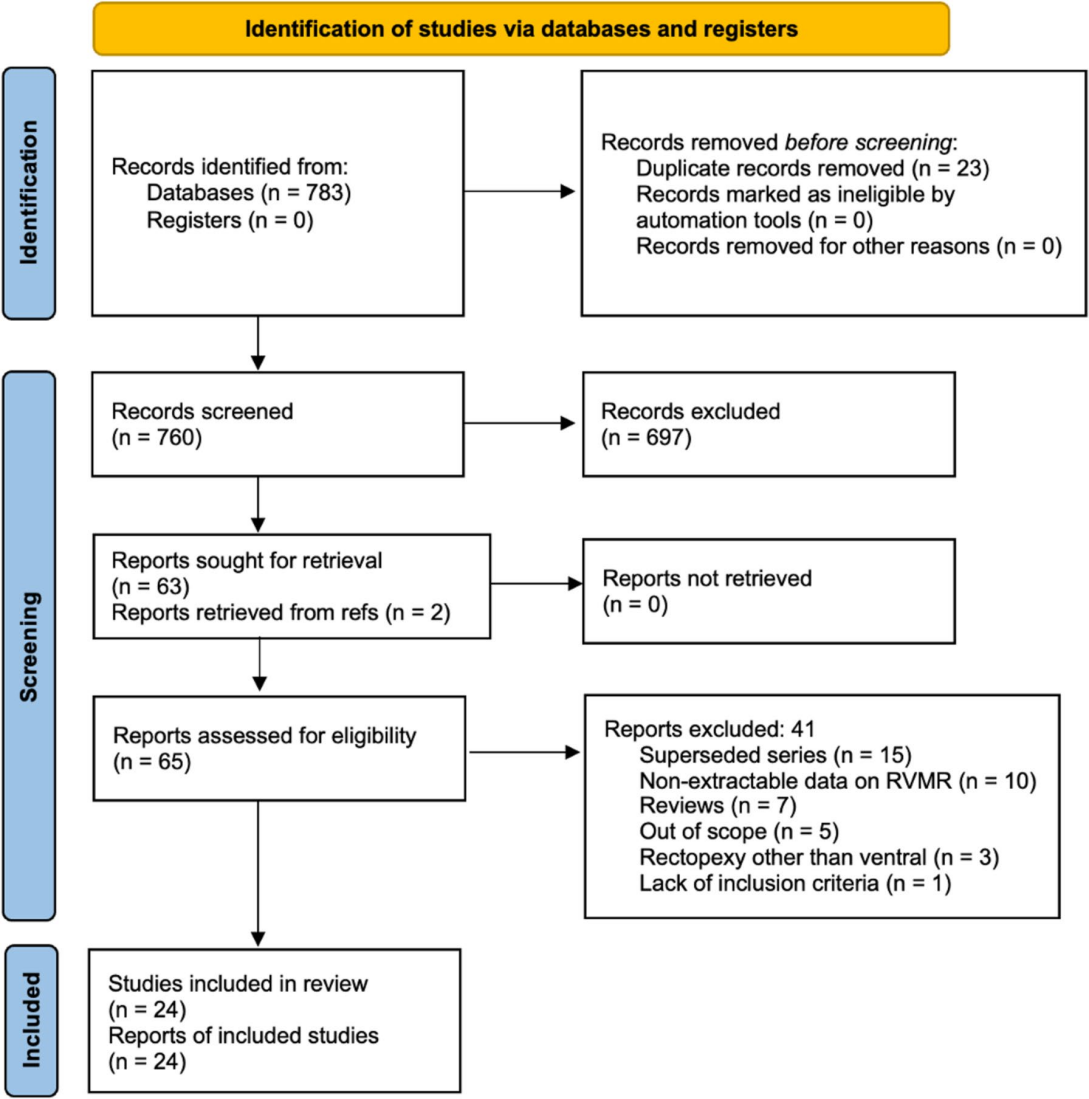
PRISMA diagram. RVMR: robotic ventral mesh rectopexy

A total of 760 unique articles were identified after the removal of 23 duplicates from an initial pool of 783 records retrieved from database searches and additional references (Fig. 1). After title screening, 574 articles were excluded, leaving 186 abstracts for further review. Following abstract screening, 123 articles were excluded, resulting in 63 full-text articles assessed for eligibility. Two additional articles were included from reference lists, bringing the total to 65 full-text articles.

Of these, 41 articles were excluded for the following reasons: superseded series (*n* = 15); no extractable data on RVMR (*n* = 10); reviews (*n* = 7); out-of-scope articles (*n* = 5); reporting combined pelvic procedures along with RVMR or other forms of rectopexy (*n* = 3); no inclusion criteria reported (*n* = 1). Ultimately, 24 studies met the inclusion criteria and were included in this systematic review (Tables 1 and 2).

**Table 1:** Studies included

First author	Year	Country	Design	Duration (months)	No. patients (% female)	Age (mean or median)	Platform	Mesh (*n*, %)	
								Synthetic	Biologic
Heemskerk [4]	2007	Netherlands	Case–control	29	12	NR	Da Vinci S	12 (100)	0
Buchs [15]	2013	Switzerland	RCS	8	3 (100)	74	Da Vinci Si	NR	NR
Mantoo [20]	2013	France	Case–control	41	44 (100)	61	Da Vinci Si	44 (100)	0
Mehmood [21]	2014	UK	PCS	38	17 (100)	61	Da Vinci X	0	17 (100)
Faucheron [9]	2016	France	Case–control	13	10 (90)	57	Da Vinci S	10 (100)	0
Al-Mazrou [17]	2017	USA	Case report	/	1 (100)	58	Da Vinci Si	0	1 (100)
Inaba [10]	2017	USA	RCS	47	24 (95.8)	67.5	Da Vinci Si	24 (100)	0
Atasoy [18]	2017	Turkey	Case report	/	1 (100)	40	Da Vinci S/Si	1 (100)	0
Carvalho [22]	2018	USA	Case–control	96	78	NR	NR	NR	NR
Brunner [23]	2018	Germany	Case–control	54	23 (100)	55.5	Da Vinci Si	0	23 (100)
Postillon [11]	2020	France	RCS	60	96 (89.6)	62.3	Da Vinci S/Si	96 (100)	0
Colucci [30]	2020	Switzerland	Case report	/	1 (100)	55	Da Vinci Xi	0	1 (100)
Ng [24]	2021	Singapore	Cross-sectional	60	13 (100)	62	NR	1 (8)	12 (92)
Van der Schans [14]	2022	Netherlands	RCS	106	273 (91.6)	58	Da Vinci Si/Xi	273 (100)	0
Laitakari [8]	2022	Finland*	Case–control	60	152 (100)	62.7	Da Vinci Si	152 (100)	0
Araujo [25]	2022	Brazil	Case report	/	1 (100)	40	Da Vinci Xi	1 (100)	0
Athanasiou [26]	2023	UK	Case report	/	2 (100)	32/NR	Da Vinci Xi	0	2 (100)
Dumas [12]	2023	France	Case–control	12	30 (96.7)	67	Da Vinci X	30 (100)	0
Zigiotto [19]	2023	Italy	Case report	/	1 (100)	56	Da Vinci Si	1 (100)	0
Bak [27]	2023	South Korea	Case report	/	1 (100)	75	Da Vinci SP	1 (100)	0
Marra [13]	2023	Italy	PCS	13	22 (95.5)	60.3	Da Vinci Xi	22 (100)	0
Drissi [28]	2023	France	Case–control	72	47 (100)	61.3	Da Vinci S	0	47 (100)
Chaoui [16]	2024	Belgium	Case–control	67	77	NR	Da Vinci Si	NR	NR
Rogers [29]	2024	USA	Case report	/	1 (100)	79	Da Vinci Xi	1 (100)	0

^*^Multicentre; *RCS*, retrospective cohort study; *PCS*, prospective cohort study; *NA*, not applicable; *NR*, not reported

**Table 2:** Inclusion criteria

First author	Year	No. patients	Anatomical abnormalities					Preoperative workup		
			ERP	IRP	Rectocele	Enterocele	Multiple	Symptom scores	Imaging	Other tests
Heemskerk [4]	2007	12	12	0	0	0	NR	NR	NR	NR
Buchs [15]	2013	3	3	0	0	0	0	NR	MRI defecography	ERUS, manometry
Mantoo [20]	2013	44	12	0	32	NR	NR	NR	NR	NR
Mehmood [21]	2014	17	17	0	0	0	NR	NR	MRI defecography	ERUS
Faucheron [9]	2016	10	8	0	0	2	0	NR	X-ray defecography	Proctosigmoidoscopy, manometry, ERUS, and colonic transit time
Al-Mazrou [17]	2017	1	0	1	0	0	NR	CCIS/KESS	NR	NR
Inaba [10]	2017	24	24	NR	NR	NR	NR	NR	NR	NR
Atasoy [18]	2017	1	0	0	0	0	1^§^	Browning & Parks; Rome II criteria	Defecography	NR
Carvalho [22]	2018	78	NR	NR	NR	NR	NR		NR	NR
Brunner [23]	2018	23	4	3	12	6	NR	NR	X-ray or MRI defecography	Manometry, colonoscopy, and colonic transit time performed selectively when indicated
Postillon [11]	2020	96	96	NR	NR	NR	NR	NR	NR	NR
Colucci [30]	2020	1	0	0	1	0	NR	NR	MRI defaecography	NR
Ng [24]	2021	13	3	6	1	0	3^§§^	NR	X-ray or MRI defecography if ERP was not clinically evident	Colonscopy; ERUS, manometry and/or transit marker studies if necessary
Van der Schans [14]	2022	273	73	200	0	0	0	0	X-ray or MRI defecography	NR
Laitakari [8]	2022	152	36	116	NR	NR	NR	NR	NR	NR
Araujo [25]	2022	1	1	0	0	0	NR	NR	NR	NR
Athanasiou [26]	2023	2	2	0	0	0	NR	NR	NR	NR
Dumas [12]	2023	30	6	0	6	0	18^§§§^	NR	X-ray or MRI defecography	Manometry in case of dyschezia or FI
Zigiotto [19]	2023	1	1	0	0	0	NR	FISI, CCIS, SF- 36	NR	NR
Bak [27]	2023	1	1	0	0	0	NR	NR	Defecography	NR
Marra [13]	2023	22	NR	NR	NR	NR	NR	NR	NR	NR
Drissi [28]	2023	47*	20	NR	26	NR	NR	NR	X-ray or MRI defecography when multicompartment disease or combined prolapse were suspected	Colonoscopy; ERUS, manometry in case of FI
Chaoui [16]	2024	77	NR	NR	NR	NR	NR	CCCS, ODS, CCIS	NR	NR
Rogers [29]	2024	1	1	0	0	0	NR	NR	NR	NR

*ERP*, external rectal prolapse; *IRP*, internal rectral prolapse; *NR*, not reported; *KESS*, Knowles-Eccersley-Scott Symptom score; *FISI*, Fecal Incontinence Severity Index; *CCCS*, Cleveland Clinic Constipation score; *ODS*, Obstructed Defecation score; *CCIS*, Cleveland Clinic Incontinence score; *ERUS*, endorectal ultrasonography; *FI*, fecal incontinence; *Indication was classified as “other” in 1 patient; ^§^IRP with rectocele and solitary rectal ulcer syndrome; ^§§^IRP + rectocele; ^§§§^IRP + rectocele (*n* = 17), ERP + rectocele (*n* = 1)

### Study characteristics

The 24 included studies comprised 930 patients who underwent RVMR. Study designs varied, including 9 case–control studies, 8 case reports, 6 cohort studies, and one cross-sectional study. The duration of the studies ranged from 8 to 106 months, with patient sample sizes ranging from 1 to 273. Most studies were single-center investigations, with only one multicenter study identified [8].

The majority of the patients were female, with a female proportion exceeding 90% in most studies. Six studies [9–14], including 37 male patients (range: 1–23 per study), reported outcomes of RVMR in males.

Patient age varied widely, with reported means or medians ranging from 32 to 79 years. Robotic platforms included Da Vinci S, Si, X, Xi, and SP systems. Of the 24 included studies, 21 reported details on the type of mesh used, covering a total of 772/930 (83%) patients. Among these, 671 patients (87%) received a synthetic mesh, while 101 patients (13%) were treated with a biological mesh.

Two studies excluded patients with a hostile abdomen, defined as a history of extensive abdominal surgery and likely multiple adhesions [4, 15].

### Preoperative workup

Preoperative imaging was described in 16 (67%) studies, with defecography (X-ray or MRI) being the most frequently used modality. In contrast, 36% of studies did not report any imaging protocols. Other preoperative assessments included endorectal ultrasonography, anorectal manometry, colonoscopy, and colonic transit studies, though these were performed selectively.

### Indications for RVMR

**Fig. 2:**
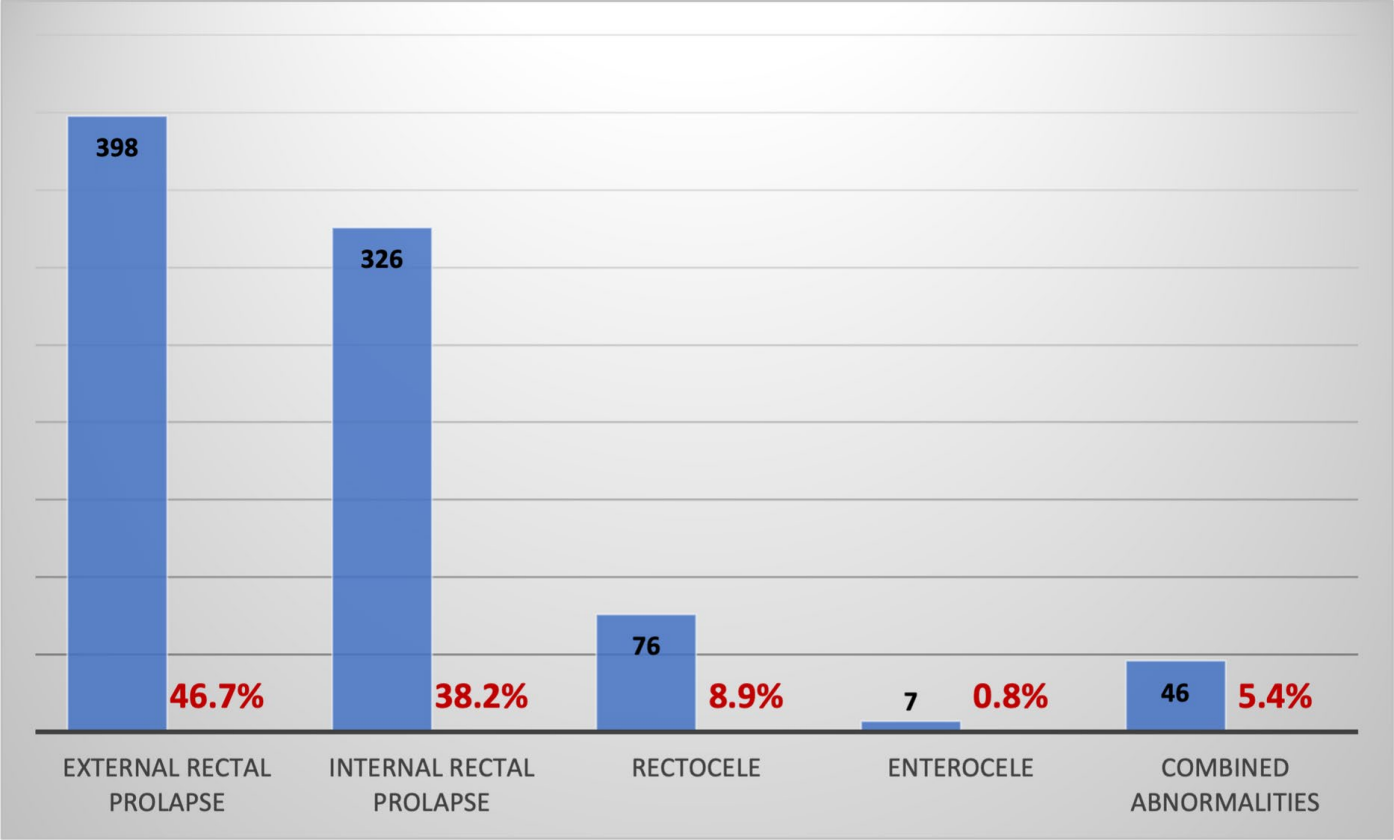
Distribution of indications among studies. Data could not be extracted in one study due to unavailability of the full-text [16]

The indications for RVMR were diverse. ERP was the most commonly reported anatomical abnormality (*n* = 398 [46.7%]), followed by IRP (*n* = 326 [38.2%]), rectocele (*n* = 76 [8.9%]), and enterocele (*n* = 7 [0.8%]). Combined abnormalities were noted in 46 [5.4%] cases (Fig. 2). Data could not be extracted in one study as the full text could not be retrieved [16].

Symptom-based indications were inconsistently reported. Only four (17%) studies employed standardized scoring systems [16–19], including the Knowles-Eccersley-Scott Symptom score (KESS), obstructed defecation score (ODS), and Cleveland Clinic scores for constipation (CCCS) or incontinence (CCIS).

#### External rectal prolapse (ERP)

Nineteen studies evaluated the outcomes of RVMR in patients with ERP, including a total of 398 patients (range: 1–96 per study) [4, 8–12, 14, 15, 19–29]. Among these, the majority were case–control studies (8), followed by case reports (5), retrospective cohort studies (4), a prospective cohort study, and a cross-sectional study. These studies consistently reported that RVMR is a safe and effective option for ERP, providing anatomical correction and functional improvement with a low complication rate.

Three case reports [19, 26, 27] documented the use of RVMR for recurrent ERP.

#### Internal rectal prolapse (IRP)

RVMR was also indicated in patients with obstructing defecation syndrome and/or fecal incontinence, presenting with IRP associated or not with other anatomical abnormalities (e.g., enterocele, rectocele), refractory to conservative management. Five studies reported outcomes of RVMR for IRP, including 326 patients (range: 1–200 per study) [8, 14, 17, 23, 24]. Among these, two were case–control studies, one cross-sectional study, one retrospective cohort study, and one case report. Functional outcomes consistently demonstrated significant improvement in obstructing defecation syndrome and fecal incontinence, particularly in patients with coexisting anatomical abnormalities, such as rectocele. Both LVMR and RVMR with biological mesh were safe and effective, with significant reductions in symptoms measured by validated scoring systems (e.g., CCCS, CCIS, and ODS), and high levels of patient satisfaction. While functional and quality of life (QoL) outcomes were generally comparable between LVMR and RVMR, some studies reported lower mid-term anal incontinence symptom scores for RVMR patients, albeit with a higher incidence of de novo pelvic pain. Technical reports of RVMR underscored its feasibility and precision in correcting multiple anatomical defects, with low complication rates and favorable short-term outcomes. One case report described a successful correction of IRP in a 58-year-old patient, with no recurrence observed at 3-month follow-up and an uneventful recovery [17].

#### Isolated rectocele

RVMR for isolated rectocele was reported in five studies, including 76 patients [12, 20, 23, 24, 28, 30]. These comprised four case–control studies, one cross-sectional study, and one case report.

The findings suggested that RVMR is a feasible and effective option for managing rectocele-related symptoms, such as incomplete evacuation, pelvic discomfort, and obstructing defecation. Additional benefits included the potential for high patient satisfaction and reduced recurrence when biologic mesh was used.

Both LVMR and RVMR with biological mesh were safe and effective in reducing symptoms associated with rectocele, including ODS. Patients also reported high levels of satisfaction.

RVMR offered advantages in addressing complex or recurrent cases. For instance, a case report described a robotic-assisted ventral re-rectopexy performed on a 55-year-old woman with a symptomatic recurrent rectocele [30]. The procedure, which incorporated the pre-existing mesh, resulted in a successful resolution of symptoms and no recurrence at 6 months follow-up.

#### Coexistence of recetocele and enterocele

One case–control study involving 32 patients evaluated the use of RVMR for rectocele associated with enterocele [20]. This study compared outcomes between RVMR and LVMR and demonstrated promising results, including satisfactory anatomical correction, functional improvement, and a lower rate of early complications following RVMR.

Both RVMR and LVMR were effective in improving obstructing defecation syndrome and fecal incontinence. However, RVMR demonstrated superior improvement in the former, particularly in terms of reduced straining, decreased need for digital assistance, and higher patient satisfaction after defecation.

In one study [20], RVMR had fewer early complications compared to LVMR (2% vs. 11%; *p* = 0.019) and resulted in significantly lower intraoperative blood loss (8 ± 34 ml vs. 42 ± 88 ml; *p* = 0.012), though requiring a longer operative time (191 ± 26 min vs. 163 ± 39 min for LVMR; *p* = 0.0002), which included robotic setup time. Improvements in sexual activity and relief from dyspareunia were observed in both groups, with no new cases of dyspareunia reported.

#### Multicompartment pelvic floor disorders

Three studies, including two case–control studies [12, 20] and one retrospective cohort study [15], explored the use of RVMR in patients with multicompartment pelvic floor disorders. These studies reported improvements in functional outcomes, including relief from obstructing defecation and fecal incontinence, as well as enhanced patient satisfaction and reduced recurrence rates.

RVMR was found to provide effective anatomical correction and functional restoration, even in complex cases involving concurrent pelvic floor abnormalities. By comparing laparoscopic and robotic approaches, Buchs et al. [15] highlighted the advantages of RVMR in terms of better satisfaction rates, reduced recurrence, and improved functional outcomes.

## Discussion

### Summary of evidence

The review highlights significant variability in indications, preoperative workup, and reporting standards for RVMR. The absence of uniform criteria for patient selection and the inconsistent use of preoperative imaging suggest a lack of standardization in clinical practice.

Our scoping review highlights several important trends in the use of RVMR. Notably, the majority of the procedures were performed in patients with ERP, a full-thickness prolapse that presents a clear and measurable anatomical abnormality postoperatively. This suggests that the adoption of robotic surgery in rectopexy has been proceeding cautiously, with a focus on “clear-cut” indications like ERP. Such cases offer more straightforward assessments of surgical success, as the anatomical resolution is easier to measure compared to high-grade IRP. It is worth noting that nearly all (316/326 [97%]) of the patients who underwent RVMR for IRP were reported in two large studies [8, 14].

IRP presents greater challenges, both diagnostically and in defining surgical outcomes. Notably, there is significant heterogeneity in the definition of “recurrence.” This term may refer to clinical or functional improvements, such as obstructed defecation syndrome or fecal incontinence, or, alternatively, to anatomical outcomes, such as the persistence or resolution of intussusception.

Several studies failed to provide detailed descriptions of the preoperative evaluation, making it difficult to ascertain whether patients met standardized diagnostic criteria for the reported anatomical abnormalities. More than one-third of studies did not report any imaging protocols, introducing a worrying selection bias that undermines comparability across studies. Moreover, the predominance of synthetic mesh use, often without a clear rationale, underscores an area for further investigation regarding material selection and its impact on outcomes.

In the two largest series, which included over 100 patients, indications for RVMR were described broadly and lacked detailed standardization [8, 14]. For instance, Van der Schans et al. [14] reported on patients with ERP or Oxford grade III/IV IRP, often associated with middle pelvic compartment descent (e.g., rectocele or enterocele), while Laitakari et al. [8] noted that the indications and follow-up protocols were determined by individual centers, emphasizing the heterogeneity in patient selection.

The complexity of redo surgery for recurrent cases further highlights the need for surgical expertise and careful patient selection.

While the evidence was limited and no studies have specifically focused on males, the available data suggested that RVMR provides comparable anatomical and functional outcomes to those reported in female patients, including low complication rates and satisfactory symptom resolution.

### Limitations

This study has several limitations. One major issue lies in the heterogeneity of reporting across the included studies. Variability in how patient characteristics, indications, preoperative assessments, and surgical techniques were documented made it challenging to compare findings or synthesize conclusions effectively. The absence of standardized diagnostic and inclusion criteria further exacerbated this variability, introducing a potential selection bias that may limit the generalizability of the findings.

To enhance the focus and comparability of our review, we intentionally excluded studies involving robotic surgery for more complex procedures, such as when RVMR was combined with sacrocolpopexy or other pelvic floor interventions. These cases often involve more intricate patient selection processes, which would have further complicated comparisons across studies. By focusing exclusively on RVMR cases, we anticipated finding greater homogeneity in inclusion criteria. However, this was not the case, as significant variability still emerged.

It is also worth noting that, to date, only one randomized controlled trial comparing RVMR and LVMR has been conducted [31]. Unfortunately, this study had to be excluded from our review as it was superseded by a subsequent publication by the same research group [8], which included the same patient cohort. Although the latter study was of lower methodological quality (i.e., a case–control design), it involved a larger sample size. Given the scope and objectives of our scoping review, we prioritized the inclusion of the broader dataset provided by Laitakari et al. [8].

The majority of studies were retrospective, with small sample sizes and limited follow-up periods. Similar methodological weaknesses were previously found in studies on laparoscopic and other rectopexy procedures [6]. Although case reports were included due to the qualitative nature of this scoping review, we acknowledge that many of them lacked adherence to the CARE guidelines, with incomplete reporting of key elements such as symptom profiles, imaging workup, or rationale for surgical indication. Their inclusion nevertheless helped to underscore the current gaps in standardized reporting and patient characterization across the literature.

Finally, 15 studies were excluded because they were superseded by subsequent publications from the same research groups. While it is common for authors to use the same patient cohorts to address different aims across studies, this practice occasionally bordered on “salami slicing,” where data from a single cohort are fragmented into multiple publications without sufficient novelty. Such practices require careful scrutiny, as they can inflate the apparent volume of research while adding limited new insights.

## Conclusions

This scoping review highlights the significant variability in clinical practice regarding indications, preoperative workup, and reporting standards for RVMR. ERP and IRP are the most commonly reported indications, but the absence of standardized selection criteria and inconsistent use of preoperative imaging underscores a critical need for better-defined protocols.

Future research should focus on (a) standardizing patient selection criteria and preoperative evaluation protocols; (b) conducting multicenter, prospective, and randomized trials to establish the role of RVMR in managing IRP, isolated rectocele, and combined pelvic floor abnormalities; and (c) Investigating the long-term efficacy and safety of synthetic versus biological mesh in RVMR.

## Supplementary Information

Below is the link to the electronic supplementary material.

Supplementary file1 (DOCX 20 KB)

Supplementary file2 (PDF 509 KB)

## Data Availability

No datasets were generated or analysed during the current study.

## References

[1] Grossi U, Heinrich H, Di Tanna GL et al (2021) Systematic characterization of defecographic abnormalities in a consecutive series of 827 patients with chronic constipation. Dis Colon Rectum 64(11):1385–1397 33833142 10.1097/DCR.0000000000001923

[2] Grossi U, Di Tanna GL, Heinrich H, Taylor SA, Knowles CH, Scott SM (2018) Systematic review with meta-analysis: defecography should be a first-line diagnostic modality in patients with refractory constipation. Aliment Pharmacol Ther 48(11–12):1186–1201 30417419 10.1111/apt.15039

[3] D’Hoore A, Cadoni R, Penninckx F (2004) Long-term outcome of laparoscopic ventral rectopexy for total rectal prolapse. Br J Surg 91(11):1500–1505 15499644 10.1002/bjs.4779

[4] Heemskerk J, de Hoog DE, van Gemert WG, Baeten CG, Greve JW, Bouvy ND (2007) Robot-assisted vs. conventional laparoscopic rectopexy for rectal prolapse: a comparative study on costs and time. Dis Colon Rectum 50(11):1825–1830 17690936 10.1007/s10350-007-9017-2 PMC2071956

[5] Keating T, Fleming CA, Brannigan AE (2022) International Robotic Rectopexy Delphi, Using a modified Delphi process to explore international surgeon-reported benefits of robotic-assisted surgery to perform abdominal rectopexy. Tech Coloproctol 26(12):953–962 35986805 10.1007/s10151-022-02679-w

[6] Grossi U, Knowles CH, Mason J et al (2017) Surgery for constipation: systematic review and practice recommendations: results II: Hitching procedures for the rectum (rectal suspension). Colorectal Dis 19(Suppl 3):37–48 28960927 10.1111/codi.13773

[7] Tricco AC, Lillie E, Zarin W et al (2018) PRISMA Extension for Scoping Reviews (PRISMA-ScR): checklist and explanation. Ann Intern Med 169(7):467–473 30178033 10.7326/M18-0850

[8] Laitakari KE, Makela-Kaikkonen JK, Kossi J et al (2022) Mid-term functional and quality of life outcomes of robotic and laparoscopic ventral mesh rectopexy: multicenter comparative matched-pair analyses. Tech Coloproctol 26(4):253–260 34935090 10.1007/s10151-021-02563-z PMC8917003

[9] Faucheron JL, Trilling B, Barbois S, Sage PY, Waroquet PA, Reche F (2016) Day case robotic ventral rectopexy compared with day case laparoscopic ventral rectopexy: a prospective study. Tech Coloproctol 20(10):695–700 27530905 10.1007/s10151-016-1518-3

[10] Inaba CS, Sujatha-Bhaskar S, Koh CY et al (2017) Robotic ventral mesh rectopexy for rectal prolapse: a single-institution experience. Tech Coloproctol 21(8):667–671 28871416 10.1007/s10151-017-1675-z

[11] Postillon A, Perrenot C, Germain A et al (2020) Long-term outcomes of robotic ventral mesh rectopexy for external rectal prolapse. Surg Endosc 34(2):930–939 31183789 10.1007/s00464-019-06851-6

[12] Dumas C, Duclos J, Le HuuNho R et al (2023) Is robotic ventral mesh rectopexy for pelvic floor disorders better than laparoscopic approach at the beginning of the experience? A retrospective single-center study. Int J Colorectal Dis 38(1):216 37589810 10.1007/s00384-023-04511-9

[13] Marra AA, Campenni P, De Simone V, Parello A, Litta F, Ratto C (2023) Technical modifications for cost optimization in robot-assisted ventral mesh rectopexy: an initial experience. Tech Coloproctol 27(7):551–557 36802041 10.1007/s10151-023-02756-8 PMC9938509

[14] van der Schans EM, Verheijen PM, Moumni ME, Broeders I, Consten ECJ (2022) Evaluation of the learning curve of robot-assisted laparoscopic ventral mesh rectopexy. Surg Endosc 36(3):2096–2104 33835255 10.1007/s00464-021-08496-w

[15] Buchs NC, Pugin F, Ris F, Volonte F, Morel P, Roche B (2013) Early experience with robotic rectopexy. Int J Med Robot 9(4):e61-65 23776088 10.1002/rcs.1498

[16] Chaoui AM, Chaoui I, Olivier F, Geers J, Abasbassi M (2024) Outcomes of robotic versus laparoscopic ventral mesh rectopexy for rectal prolapse. Acta Chir Belg 124(2):91–98 36905354 10.1080/00015458.2023.2191073

[17] Al-Mazrou AM, Kiran RP, Pappou EP, Feingold D, Lee-Kong S (2017) Robotic ventral mesh rectopexy - a video vignette. Colorectal Dis 19(7):695 28520090 10.1111/codi.13736

[18] Atasoy D, Aghayeva A, Bayraktar O et al (2017) Robotic ventral mesh rectopexy technique for rectal intussusception with rectocele - a video vignette. Colorectal Dis 19(10):947 28816010 10.1111/codi.13847

[19] Zigiotto D, Sturiale A, Naldini G (2023) Robotic rectosigmoidopexy for recurrent external prolapse after altemeier procedure. Dis Colon Rectum 66(12):e1264 37646675 10.1097/DCR.0000000000002932

[20] Mantoo S, Podevin J, Regenet N, Rigaud J, Lehur PA, Meurette G (2013) Is robotic-assisted ventral mesh rectopexy superior to laparoscopic ventral mesh rectopexy in the management of obstructed defaecation? Colorectal Dis 15(8):e469-475 23895633 10.1111/codi.12251

[21] Mehmood RK, Parker J, Bhuvimanian L et al (2014) Short-term outcome of laparoscopic versus robotic ventral mesh rectopexy for full-thickness rectal prolapse Is robotic superior? Int J Colorectal Dis 29(9):1113–1118 24965859 10.1007/s00384-014-1937-4

[22] Carvalho ECME, Hull T, Zutshi M, Gurland BH (2018) Resection rectopexy is still an acceptable operation for rectal prolapse. Am Surg 84(9):1470–1475 30268178

[23] Brunner M, Roth H, Gunther K, Grutzmann R, Matzel KE (2018) Ventral rectopexy with biological mesh: short-term functional results. Int J Colorectal Dis 33(4):449–457 29442156 10.1007/s00384-018-2972-3

[24] Ng YY, Tan E, Fu CWP (2022) Trends in the surgical management of rectal prolapse: an Asian experience. Asian J Endosc Surg 15(1):110–120 34448361 10.1111/ases.12978

[25] Araujo SEA, Seid VE, Portilho AS et al (2022) Robotic ventral mesh rectopexy. Dis Colon Rectum 65(1):e4 34882635 10.1097/DCR.0000000000002073

[26] Athanasiou C, Dowsett D, Shaikh I (2023) Comparison of a primary and a recurrent case of full rectal prolapse treated with robotic ventral mesh rectopexy - a video vignette. Colorectal Dis 25(2):331–332 36000292 10.1111/codi.16307

[27] Bak MR, Lee IK, Lee YS, Lee CS (2023) Single-port robotic ventral mesh rectopexy for recurrent rectal prolapse with the da Vinci SP platform: a video vignette. Asian J Surg 46(3):1317–1318 36123210 10.1016/j.asjsur.2022.08.092

[28] Drissi F, Rogier-Mouzelas F, Fernandez Arias S, Podevin J (2023) Meurette (2023) Moving from laparoscopic synthetic mesh to robotic biological mesh for ventral rectopexy: results from a case series. J Clin Med 12(17):5751. 10.3390/jcm12175751 37685818 10.3390/jcm12175751 PMC10488879

[29] Rogers P, Dourado J, Delgado Z, Strassmann V, Vogler S, DaSilva G (2024) Robotic ventral mesh rectopexy: troubleshooting in redo surgery - a video vignette. Colorectal Dis 26(8):1646–1647 38863117 10.1111/codi.17070

[30] Colucci N, Abbassi Z, Toso C, Hahnloser D (2020) Robotic ventral re-rectopexy for symptomatic rectocele recurrence - a video vignette. Colorectal Dis 22(11):1760 32400019 10.1111/codi.15117

[31] Makela-Kaikkonen J, Rautio T, Ohinmaa A et al (2019) Cost-analysis and quality of life after laparoscopic and robotic ventral mesh rectopexy for posterior compartment prolapse: a randomized trial. Tech Coloproctol 23(5):461–470 31069557 10.1007/s10151-019-01991-2 PMC6620369

