# Supplementary material for: Scoping review of indications for robotic ventral mesh rectopexy: addressing variability in patient selection
Source: Int J Colorectal Dis. 2025 Apr 25;40(1):102. doi: 10.1007/s00384-025-04893-y (PMC12021707; doi:10.1007/s00384-025-04893-y)
Supplement: Supplementary file 1 — Supplementary file1 (DOCX 20 KB) [file 384_2025_4893_MOESM1_ESM.docx]

**Appendix 1.** Search strategy

**Pubmed:** ((Robotic Surgical Procedures OR Robotics[MeSH Terms])) AND (((((Rectal Prolapse[MeSH Terms]) OR (Rectocele[MeSH Terms])) OR (intussusception[MeSH Terms])) OR (Pelvic Floor Disorders[MeSH Terms])) OR (Rectum[MeSH Terms]))

**Scopus and Web of Science:** ((Robotic AND surgical AND procedures OR robotics[all AND fields])) AND (((((rectal AND prolapse) OR (rectocele)) OR (intussusception)) OR (pelvic AND floor AND disorders)) OR (rectum))
